# Gender Differences in Subjective Pain Perception during and after Tattooing

**DOI:** 10.3390/ijerph17249466

**Published:** 2020-12-17

**Authors:** Joanna Witkoś, Magdalena Hartman-Petrycka

**Affiliations:** 1 Faculty of Medicine and Health Science, Andrzej Frycz Modrzewski Krakow University, G. Herlinga-Grudzińskiego Street 1, 30-705 Kraków, Poland; 2 Department of Basic Biomedical Science, Faculty of Pharmaceutical, Sciences in Sosnowiec, Medical University of Silesia, Kasztanowa Street 3, 41-200 Sosnowiec, Poland; mhartman@sum.edu.pl

**Keywords:** gender differences, pain, tattoo, time of tattooing, bleeding, level of stress

## Abstract

Background: The aim of the research was to analyse the impact of gender on pain perception during and directly after tattooing, with the following predictors as covariates: the body area chosen for a tattoo, the character of the pain, the time it takes to complete the tattoo, bleeding, the level of stress, analgesics taken before the tattooing procedure, and the cycle phase. Methods: A total of 1092 participants took part in this study (F: 863, M: 229). A proprietary survey was used in the research, including patient characteristics and questions relating to the above-mentioned variables. Multiple regression analyses were used for continuous outcomes and multiple logistic regression analyses for binary outcomes. Results: Factors increasing pain during tattooing include: time B: 0.35; 95% CIs: 0.27–0.43; *p* = 0.001; bleeding B: 0.36; 95% CIs: 0.00–0.72; *p* = 0.052; level of stress B: 0.45; 95% CIs: 0.31–0.60; *p* = 0.001; pain medications taken before tattooing B: 1.42; 95% CIs: 0.60–2.23; *p* = 0.001. Factors increasing pain after tattooing include: time B: 0.21; 95% CIs: 0.15–0.27; *p* = 0.001; bleeding B: 0.47; 95% CIs: 0.20–0.72; *p* = 0.001; level of stress B: 0.15; 95% CIs: 0.04–0.26; *p* = 0.001. Conclusions: There was no difference between females and males in pain intensity during tattooing. Directly after the procedure, however, pain intensity was higher in women when compared to men. The most important factors increasing pain were time, bleeding, and the level of stress.

## 1. Introduction

Tattooing, which involves creating a permanent skin pigmentation, has become a common phenomenon that has evolved from a cultural taboo related to ostracizing tattooed individuals into a way of expressing oneself and a sign of individualism [1,2]. Tattoos can be a form of body adornment, reflect works of art, or be a fashionable accessory that draws attention and definitely helps one to stand out in society. There are numerous motivations to get a tattoo. It can be a marker of individuality, personality, and independence, or, conversely, a sign of identification with a particular group, being a sign of one’s sense of belonging to a given subculture [3,4,5,6]. Numerous authors believe that regardless of the above-mentioned motivations to get a tattoo, the ultimate evolutionary aim is an impulse rooted in sexual selection and the need to increase an individual’s attractiveness on such a basis [7,8,9]. A tattoo has a relatively permanent character, and its creation involves a painful sensation of multiple skin punctures performed with a needle. On the one hand, a tattoo is a visual sign of rejecting a taboo; on the other, however, it cannot be overlooked that it is an act of self-injury inextricably linked with pain [2,10].

Pain is a complex process that involves the integration of sensory and emotional information. Experiencing pain is linked to both the action of an injuring stimulus that affects tissues and the awareness emerging based on the mental interpretation of those occurring phenomena, additionally modified by past experiences and psychosomatic predispositions. The awareness of pain occurrence triggers a whole chain of reactions and methods of pain response, from proper recognition of the pain stimulus to appropriate emotional assessment. All painful experiences trigger emotional reactions, as information about pain is transmitted to the limbic system [11].

The unpleasant pain sensation during tattooing is linked to the presence in the human skin of free nerve endings, nociceptors, reacting to tissue damaging stimuli, which in the case of this procedure are understood as the piercing of the skin with a needle and the mechanism of pain sensation. This sensation makes an individual aware of the pain and enables them to subjectively assess it and undertake preventive action [8,12]. The pain experienced during tattooing is a receptor (nocireceptor) pain due to its pathomechanism. More precisely, it is one of the types of receptor pain, i.e., somatic pain, caused by an injury to soft tissue, including the skin. This pain is of a nature that is relatively easy to describe and localise, and this distinguishes it from receptor pain of the second type, i.e., visceral pain, as well as from non-receptor (neuropathic) pain [11,12,13].

Experiences that accompany humans throughout their lives, especially pain, are linked to various external and internal factors, and the determinants of pain that may impact its sensation, interpretation, and modification. Due to this, pain intensity and quality are subject to various conditions. The same stimulus can be perceived differently by various—or even the same—people in diverse external conditions, somatic and mental states. Pain perception, a sensual impression based on the subjective assessment of a stimulus affecting the human body, depends on many factors, such as sociological and cultural factors (age, race, family history), psychological (personality traits, fear, depression, cognitive factors) and biological factors, such as gender, genetic and hormonal predisposition, or the activity of endogenous opioids [11,12,13]. All of the above-mentioned factors significantly contribute to the difference in pain perception among people. However, there is an ever-increasing interest in identifying new components that may contribute to the explanation of changes in pain perception, especially considering the gender factor.

In recent years, the particular role of gender in the perception of individual perception of pain has been noted. For many years, clinical trials more often involved male subjects than females due to hormonal changes related to women’s menstrual cycle, which may potentially significantly influence the perception of pain [14]. Numerous studies have shown gender-related differences in response to various experimental pain stimuli, including mechanical, electrical, thermal, ischemic, chemical or pathological pain [15,16,17,18,19,20]. It has been shown that women have a lower pain threshold and a lower tolerance than men to most types of nociceptive stimuli; therefore, women report increased pain sensation and further greater sensitivity to both clinical and experimental pain [21].

Individual differences influencing the perception of the pain stimulus definitely involve hormonal balance in females and males. The available scientific literature yields scarce and ambiguous—or even conflicting—views on the influence of hormonal changes occurring in females during consecutive menstrual cycle phases on the perception of an external stimulus. While some authors [22] claimed not to note any impact of hormonal changes during given menstrual cycle phases on the perception of pain stimuli in women, others [23,24] stated clearly that changing hormonal balance, including in particular current oestrogen and progesterone levels, does have an influence on stimuli perception, particularly in the follicular phase preceding ovulation [25,26]. There is also research that shows that healthy women experience pain more intensely in the luteal phase, just before menstruation. Due to their influence on brain functioning and development, oestrogens fundamentally impact physiological and behavioural functions. They may react with neuroprotective intracellular signalling pathways and they are responsible for the regulation of water-electrolyte balance, water and sodium retention and show an impact on adipose tissue. There is definitely a link between the oestrogen level and pain perception. Oestradiol is also known to affect neurotransmission and synaptic plasticity [27,28]. When investigating pain perception in females, it should be noted that hormonal changes not only occur throughout a woman’s life (puberty, pregnancy, menopause), but, in women of childbearing age, these changes take place every month and are linked to changing menstrual cycle phases: follicular and luteal [13,29]. In the case of males, testosterone levels do not decrease until senescence. The follicular phase begins on the 1st day of menstrual bleeding, which is thus the 1st day of the cycle, and ends with ovulation, which is characterised by an increased secretion of oestrogen, follicle-stimulating hormone, and luteinizing hormone just before ovulation. The luteal phase, on the other hand, follows the ovulation and is characterised by rising progesterone and oestrogen levels, with progesterone levels exceeding those of oestrogen [30].

This research undertakes to assess the differences in pain intensity in males and females during tattooing and directly upon the completion of the procedure. Moreover, the authors inspected the continuity of pain or its increasing nature, radiating to body areas other than the tattooed place, as well as bleeding during the procedure. The aim was to provide a wider analysis of gender differences regarding the age of tattooing, location and duration of the procedure, stress preceding tattooing and intake of analgesics before the appointment. An additional objective involved the analysis of pain sensation in connection with particular phases of the menstrual cycle in females, which also alter the hormonal balance and various factors independent of gender that may impact the severity of pain during and after tattooing.

## 2. Materials and Methods

The study involved 1092 participants, consisting of 863 women (79%) and 229 men (21%). The research was conducted at professional tattoo studios in Katowice, Silesian Voivodeship, as well as nationwide in Poland with the use of an online survey. The research lasted one year, from June 2019 to June 2020. At first, it was conducted at the Horkruks Tattoo studio in Katowice and other partner studios. However, after 5 months it was noticed that the previous recruitment method was ineffective, mainly due to the reluctance of the tattooed persons to complete the survey following a one-hour or longer painful tattoo session. The persons reported fatigue and a need to rest. Using this method, about 60 females and 10 males were surveyed, with about 30 persons refusing to complete the questionnaire. Subsequently, in November 2019, the authors contacted the Bioethics Committee again, asking for a change in the recruitment method from a direct survey into an online survey. The author of the project ensured full anonymity of the surveys. It was created in a digital form and sent to various Polish groups associated with people with a passion for body adornment. The link to the survey included its description with a special annotation to female participants. Only those females who were tattooed during the same month as they were completing the survey were asked to submit the questionnaire. The authors aimed to precisely define the day of a woman’s menstrual cycle when she was tattooed. The menstrual cycle was understood as repetitive, monthly hormonal changes in the female body, aimed at preparing the oocyte for fertilisation or leading to the shedding of the uterine lining in cases when no fertilisation occurred [30].

A proprietary survey was used in the research, which included questions regarding parameters such as the age, height, body weight and education of the participants. Subsequent questions regarded maximum subjective pain perception during and after the completion of the tattoo, tattooed body area, the time required to complete the tattoo and the character of experienced pain, including the potential increase in pain intensity occurring with the passing of time during the procedure and pain radiating to further body areas, other than the one affected directly by the procedure. Moreover, the participants were also asked about their pain experienced prior to the procedure and the use of analgesics. Females were also asked about the day of the menstrual cycle on which they were tattooed. Due to significant hormonal differences, menstruation was isolated from the follicular phase into a separate stage. Females were asked to provide the day of the procedure, counting from the last day of menstrual bleeding.

In order to assess the pain, the Numerical Rating Scale (NRS) was used, which comprised 11 degrees of pain intensity from 0 to 10, where 0 indicated a total lack of pain and 10 marked the highest degree of pain sensation, meaning the most severe pain the respondent has ever felt in their life. The participant was asked to select a number, which according to their subjective assessment, reflected the maximal pain occurring both during and directly after the procedure.

Inclusion criteria included: a lack of any pain on the day of tattooing, including, e.g., headache or menstrual pain. No regular use of any over-the-counter medicines, including analgesics, antihistamines, and anti-inflammatory drugs. No consumption of alcohol and energy drinks 24 h prior to the research. In females, additional inclusion criteria included: regular menstrual cycles, understood as occurring every 28 to 32 days and not using hormonal contraception. Exclusion criteria included persons with sensory disorders and chronic pain. In the case of females, additional exclusion criteria included: irregular menstrual cycles of undetermined cause and female diseases that could negatively impact normal menstrual cycle, including hormonal disorders diagnosed by a doctor. Out of all submitted surveys, around 170 were removed from further analysis. Most often, these were completed by women with irregular menstrual cycles or using hormonal contraceptives. A few surveys were also completed incorrectly and did not include the necessary data.

### Statistical Analyses

All analyses were performed using SPSS 21 software.

Gender differences were calculated for dependent variables as well as for predictors. Given the large difference in the sample sizes of men and women and the fact that the assumption of homogeneity of variances was not met in some cases, nonparametric Mann–Whitney U tests were used to determine gender differences for continuous variables. For categorical variables, chi-square tests were used to analyse gender differences. In the case of significant results for multicategorical variables, additional z tests were performed to determine which category is different between men and women.

To analyse the impact of predictors (gender, age, BMI, education, body area, time, bleeding, level of stress and analgesics taken before the procedure), a series of multivariate regression analyses were performed. For the dependent variables—NRS during tattooing, NRS after tattooing, and increasing pain—multiple linear analyses were performed, in the case of which the following parameters were reported: multiple determination coefficients (R^2^), which indicate the percent of the variance of the dependent variable explained by all predictors, and standardised regression coefficients (betas), indicating the strength of association between a given predictor and the dependent variable. In the case of categorical predictors, *p* values for differences between groups were reported. In addition, *p* values for all parameters were calculated, together with confidence intervals generated by bootstrapping in order to avoid problems with the distribution of errors and other assumptions of multiple regression analyses.

For the binomial dependent variables, namely pain during tattooing and radiating pain, multiple logistic regressions were calculated, with odds ratios and confidence intervals for each predictor. The second set of the above analyses included the cycle phase and excluded gender.

The research protocol was reviewed and approved by the Bioethical Committee of the Andrzej Frycz Modrzewski Krakow University (Permission number KBKA/58/O/2019).

## 3. Results

The descriptive results for the dependent variables and predictors are presented in Table 1.

There were significantly more women than men in the sample. In the case of dependent variables, in univariate analyses, NRS after tattooing proved different between men and women, with women declaring higher levels of pain. Increasing pain was more frequently present in the case of men. As for predictors, men were significantly older and had higher BMIs than women. Education varied between genders, with men having a vocational education more often than women and graduating high school less often. Body area proved different between genders: the upper extremity was chosen more often in the case of men, and the torso was more often chosen among women. The time required to complete the tattoo was generally longer in the case of men. Bleeding occurred more frequently in men. Women experienced more acute stress in comparison to their male counterparts.

### 3.1. Analyses with Gender and without Cycle Phase

For the dependent variable “NRS during tattooing”, there were five statistically significant predictors: education, time, bleeding, stress level and analgesics taken before the procedure (Table 2). All of them were positive, that is: the higher the education, the time of the tattooing, and the level of stress, the higher pain was measured by NRS during tattooing. In addition, the presence of bleeding and taking medication before the procedure increased the pain.

For the dependent variable “NRS after tattooing”, the following predictors were significant: gender (with women experiencing higher pain), the time required to complete the tattoo, bleeding, stress level. All of them were positive. Time required to complete the tattoo, the presence of bleeding and stress level also constituted significant positive predictors for “Increasing pain”. The same was true for the dichotomic dependent variable “Pain during tattooing”.

In the case of “Radiating pain”, a longer time of tattooing increased its presence. In addition, the categorical predictor “Body area” was significant for this dependent variable: radiating pain was more often present in the case of an upper extremity in comparison with a lower one. When compared to the torso, radiating pain was also more often present in the case of an upper extremity.

The results of multiple comparisons in the case of the categorical variable body area, for the dependent variable radiating pain, are as follows: the differences between the categories upper extremity and lower extremity was statistically significant (*p* = 0.003), with radiating pain occurring more often in the upper extremity than the lower one (83% vs. 72%). In addition, the pain sensation in the upper extremity differed from the one affecting the torso (83% vs. 76%; *p* = 0.031).

### 3.2. Analyses with Cycle Phase and without Gender

In the second set of analyses, the results were similar. Education, the time required to complete the procedure, and stress level (but not bleeding) were all positive predictors of NRS during tattooing (Table 3). The same predictors, but this time including bleeding, were significant positive predictors of NRS after tattooing. For the dependent variable “Increasing pain”, the time required to complete the procedure, bleeding, and analgesics taken prior to tattooing constituted significant positive predictors. As for “Pain during tattooing”, its presence was again positively predicted by the time required to complete the procedure, bleeding, and stress level. Finally, the presence of “Radiating pain” was negatively predicted by age (the older the participant, the rarer radiating pain was), and positively by the time required to complete the procedure.

## 4. Discussion

Body tattooing is a form of body adornment, standing out in society or seeking a symbol of individuality. The conducted research has shown that after the tattooing procedure, females experienced pain more acutely than males. However, no statistically significant differences in pain intensity were found between men and women. This could, however, be caused by the fact that most women had chosen rather small tattoos, thus requiring a shorter time to complete, while men had decided on larger designs, in which case the procedure was longer. A certain type of “equalization” of pain experiences could have occurred, as the duration of tattooing might have impacted it. Females experienced a relatively short and strong pain, using a scale to determine their subjective impression. Since the procedure was longer and the pain increased with every passing hour in male participants, they had the impression that the pain is comparable to the one experienced by their female counterparts. Differences in the type of experienced pain described by the respondents could also be associated with different body regions selected by the participants for their tattoo. The areas more frequently selected for tattooing were the torso in the case of women, and an upper extremity in the case of men. A different body region also means a different thickness of the skin and a different density of nerve endings, and these factors may contribute to differences in experiencing and describing pain.

Inter-individual variations that impact the way we experience pain stimuli, understanding the complex nature of pain, and personal differences that may influence nociceptive processes are still of interest to scientists. However, despite noticing the influence of gender that determines the personal perception of pain, some authors claimed that differences in susceptibility to pain between genders are scarce, while others highlighted their importance [14,15,16,17,18,19,20]. It is commonly known that men and women differ in terms of perceiving and experiencing pain. In the general population, women report more severe pain, and experience a greater increase in intensity, frequency and duration of the condition. The research by Klatzkin et al. [22] claimed that females were significantly more sensitive to cold, thermal, and ischemic pain than their male counterparts. According to Mencke et al. [21] in comparison to men, women reported a larger number of painful symptoms, higher pain assessment and pain-related disability. They also contacted doctors more often due to pain complaints. Research results included in this article only partially confirm the above remarks.

The conducted research did not show a statistical significance of the influence of the menstrual cycle phase in women on the perception of external stimulus, in this case puncturing their skin during tattooing. This could stem from the fact that menstrual cycle phases were divided only on a basic level, into three stages. Moreover, differences in cycle length and the length of particular cycle phases occur even in women who menstruate regularly. Mistakes could have occurred, even though only females with regular menstrual cycles were chosen for the study. Such a result, however, inspires further research and more detailed division into menstrual cycle phases and even the performance of hormonal tests. Such tests will enable precise determination of the menstrual cycle phase and give the opportunity to establish the current level of sex hormones and the exact day of ovulation.

Pain is an intrinsic element of body tattooing, as puncturing human skin with needles is a painful experience. It stems from both anatomical and physiological features of skin structure and the transmission of pain impulses from the receptor to the cerebral cortex. Despite the fact that during the procedure the skin is punctured at a quite shallow level, it is deep enough to stimulate nociceptors. A quite startling result of our research was the fact that around 10% of people in both study groups claimed that receiving a tattoo did not cause them any pain. While it is clear that every human being has a different pain threshold, which is affected by a plethora of factors that have already been mentioned here, as well as the fact that every person experiences pain in a personal way, it is challenging to explain such a result in a straightforward way. These participants might have had a particularly high pain threshold, the density of pain receptors was lower within the affected area or the skin was thicker, as this value may vary from 0.5 to 1.0 mm. The tattooed area might not have had contact with a bone, and adipose tissue may have created enough of a buffer between the needle and the nociceptor. Given the fact that the human brain interprets information about pain and compares it to former painful sensations and experiences, the brain itself could have acted as a “suppressant” to the actual interpretation of pain [12,31]. Maybe this information could provide answers allowing us to explain the possible “lack of pain” in a small percentage of participants. In these respondents, a certain type of “adjustment” could have occurred which involved long-lasting tonic pain and a stimulus acting with a fixed frequency. It is known, however, that in the case of pain, the opposite phenomenon occurs, namely hyperalgesia. Such a pain response occurs during the prolonged impact of damaging stimulus on tissues, which initiates a range of pathological processes in nociceptors, including an increased response to pain stimulus. The conducted research also highlighted this phenomenon, as pain intensity depended on the time required to complete a tattoo, as the longer the process took, the more severe the pain was. Obviously, the phenomenon of allodynia, i.e., lowering the pain threshold, is also known in medicine, albeit as a pathological one [19,21].

Another attempt at explaining the “lack of pain” in a small number of participants could encompass their mental attitude towards the process itself. For them, getting a tattoo could have been a certain kind of sacrifice and involved particular engagement in the procedure to achieve a higher idea. Pain was accepted to such an extent that it was mentally “blocked out” and made “non-existent”. There is also a group of people who highlight their resilience to pain by speaking about a “lack of pain sensation”. By doing so, they present themselves as stronger and healthier than the rest of society, pointing out their evolutionary superiority, including when presenting themselves as the right choice while choosing a life partner. Maybe exactly this mechanism was triggered among graduates of junior high, who declared lower pain intensity than those with higher education?

A phenomenon of not experiencing pain during the tattooing procedure may also be interpreted through the human ability to achieve certain states of mind during which the human brain is able to alleviate the pain [32,33]. Here we can consider possible cases of not feeling pain during hypnosis, trance-like states, or cultural procedures. Every man is able to enter light daily trances when focusing on one specific activity. A light trance can be deepened by purposefully adding to it deeper breathing, techniques supporting the attainment of tranquillity and calm, self-hypnosis, or relaxation techniques combined, for example, with appropriate music. The authors of this study do not know whether the respondents used any relaxation techniques or self-hypnosis during tattooing and whether this helped them not to feel the pain; however, a need to consider this issue stems from the obtained study results. The literature describing the use of trances and hypnosis in minor dermatological procedures showed that, for example, suggestions made during the trance helped to reduce pain in the skin and positively influenced diseases with a psychosomatic background. The relaxing reaction developing during the trance, hypnosis, or self-hypnosis certainly leads to a reduction in the experienced situational stress and pain, when it is associated with a given event. During deep relaxation, the brain temporarily “blocks” sensory information coming from the environment, including pain. According to Shenefeld [33], in the majority of people, a hypnotic suggestion relieves pain, regardless of the type of pain they are experiencing.

Even though the process of tattooing is generally linked with pain, it is not recommended to take analgesics prior to the procedure, as it has been found that such medicines may increase bleeding [34,35] during the tattooing. Participants undergoing the procedure were informed about the possible effects of analgesics at tattoo studios. Despite receiving the aforementioned information, 33% of participants—most probably due to the stress accompanying possible pain during tattooing, had decided to take analgesics prior to the procedure. Contrary to expectations, it was noted that the intake of analgesics increased the pain. Such an unforeseen effect may be explained by the fact that usually it is persons with a low pain threshold that take such medicines, with their sensitivity to pain overpowering the effects of analgesics. It is suspected that the participants most often resorted to widely available paracetamol and ibuprofen. The authors, however, do not have detailed information on pain medication taken by the participants prior to the procedure.

The results show that stress experienced before tattooing is an important factor increasing the pain sensation both before and after the procedure and that it favours pain escalation during tattooing. This is consistent with Peters’ [36] observations, who presented in his review article that negative emotions, such as fear, intensify pain sensation, while positive ones foster a decrease in received pain stimuli. Certainly, pain is definitely not merely a simple registration of a nociceptive stimulus. It should always be treated as a complex experience with a strong component of psychological factors, which are unique to every human.

Tattooing is always accompanied by bleeding; 69% of persons observed bleeding due to the performance of a tattoo, while the remaining participants reported bleeding so light that it was unnoticed. Bleeding is linked with an increase in pain intensity both during and after tattooing. This connection might be caused by the depth of punctures and/or skin properties, including the thickness of particular skin layers.

The occurrence of pain radiating to body parts other than the tattooed area is not dependent on the presence of bleeding and stress; it does, however, depend on the location of the tattoo. Radiating pain is more common in the upper extremities than the lower extremities, and also upper extremities differed from the torso. Further research is required to prove which location-related factor determines such spreading of pain. It can be presumed, however, that the density of nerve endings within a particular body area is of particular importance.

During our research, we attempted to determine the degree of pain perception in males and females undergoing the process of tattooing. Moreover, we strived to assess whether factors associated with certain individual characteristics and the specificity of the procedure impact the modification and interpretation of painful experiences. During the research, however, we encountered certain limitations. Scholars sharing our scope of academic interest should pay attention to them and shape the future direction of scientific research within these areas. Statistically significant differences in the number of tattooed men and women, their age and education were found. Unfortunately, the method of recruitment does not allow us to determine how this effect depends on the actual phenomenon of tattooing in Poland and to what extent it depended on the willingness to complete the survey in groups of males or females of different ages and education. The participants were not asked whether this was their first or subsequent tattoo. Some of the respondents might have been so-called “collectors” [37], which could have also impacted the mental interpretation of pain perception due to former experience with this type of pain, yielding a different attitude towards its “reception”. One significant limitation of the conducted study was the fact that the tattooed persons completing the online survey questionnaire did not complete it immediately after the end of their tattooing procedure. However, in the introduction to the online survey questionnaire, it was indicated that the period of time between the day of tattooing to the questionnaire completion day should not exceed one month. This was also associated with the menstrual cycle in women. It was decided that this was a maximum time for recalling the pain experienced during the procedure. The authors of the study do not know the exact time between the tattooing and the completion of the questionnaire by the respondents completing the online questionnaire, and in our opinion, this is also one of the limitations of this study.

The participants’ marital status was not revealed. Such knowledge would not influence the general results, but it would enrich the description of the study group and show whether it impacts the frequency of tattooing in society. The respondents’ motivation for getting a tattoo was also unknown. It could possibly provide an explanation for a “lack of pain” declared by a certain percentage of those tattooed. The size of the performed tattoo was unknown. This information does not significantly influence the results, as each participant was surveyed only once. Even if their tattoo required a few sessions to complete, the survey was completed only once. The time required to complete the tattoo or its part on a given day was known, which enabled the authors to compare the level of perceived pain to the duration of the procedure. This seems to be an important aspect of the research which also influenced the results.

The presented research raises issues that may be of interest to specialists representing various scientific disciplines. The publication is interdisciplinary, thus setting new directions for exploring knowledge in various areas. It raises numerous issues within the field of psychology, such as a person’s motivation to have a tattoo, tattoo symbols, concerns regarding experiencing stress prior to the procedure and individual sensitivity to pain linked with the mental attitude towards the process of tattooing. Subsequently, it also presents sociological concerns, including the education of the tattooed persons, their age, gender, material and marital status, occupation and so on. The publication can also serve as a stimulus for further exploration of medical issues, including a comparison between the overall health condition and pain experienced by a person undergoing the procedure, investigating skin properties of the tattooed person (hydration, firmness, stratum corneum thickness) and pain experienced during tattooing as well as the durability of the tattoo. In future research, it would definitely be beneficial to perform laboratory tests and determine the level of hormones, namely oestrogen and progesterone in female participants and testosterone in males, and subsequently assess the level of experienced pain. The assessment of the tattooing technique also proves to be an interesting issue. The question is whether it can in some way reduce painful experiences, for example, by choosing a given type of tattoo machine, particular needle thickness, skin penetration depth and tattoo ink types. Eventually, it should be investigated whether topical anaesthesia methods used in cosmetics and aesthetic medicine might be recommended and effective in the process of tattooing, and assess their impact on reducing pain, bleeding, and tattoo quality.

The results of the study are based on the survey, which demonstrates the opinion of all respondents willing to participate in the study. In the case of each of the above-mentioned problems, one might attempt to make them objective, apply appropriate measuring tools in reference to, e.g., the same body area being tattooed, and employ a targeted, randomised group selection in order to fully explain these compelling issues. The subject of such research would certainly find many supporters, especially given the fact that tattoos have become a popular body adornment method.

## 5. Conclusions

1. After being tattooed, women experienced more intense pain than men. However, no differences in pain perception were noted during the procedure. Compared to men, female participants reported higher stress before the procedure, but they also more frequently decided to tattoo their torso, while their tattoos required less time to complete and caused lighter bleeding.
2. The factors that most significantly increased pain perception during the process of tattooing included: the time required to complete the tattoo, the presence of bleeding, stress, and the intake of analgesics.

## Figures and Tables

**Table 1:** Descriptive results for predictors and dependent variables and gender differences.

		Total	Men	Women	*p*
Dependent variables					
NRS during tattooing		4.35 (2.60)	4.48 (2.54)	4.31 (2.61)	0.359
NRS after tattooing		2.07 (2.02)	1.76 (1.74)	2.15 (2.08)	0.028
Increasing pain	Definitely not	72 (6.59)	7 (3.40) ^a^	65 (8.50) ^b^	0.021
	Rather not	218 (19.96)	41 (19.90) ^a^	177 (23.14) ^a^	
	Rather yes	370 (33.88)	79 (38.35) ^a^	291 (38.04) ^a^	
	Definitely yes	311 (28.48)	79 (38.35) ^a^	232 (30.33) ^b^	
Pain during tattooing		971 (88.92)	206 (89.96)	765 (88.64)	0.574
Radiating pain		218 (19.96)	52 (25.24)	166 (21.70)	0.279
Predictors					
Gender		1092 (100.00)	229 (21.0)	863 (79.0)	<0.001
Age		25.19 (6.04)	27.38 (6.79)	24.61 (5.69)	<0.001
BMI		27.34 (5.14)	28.95 (5.10)	26.91 (5.07)	<0.001
Education	Junior high	81 (7.42)	15 (6.55) ^a^	66 (7.65) ^a^	0.024
	Vocational school	98 (8.97)	30 (13.10) ^a^	68 (7.88) ^b^	
	High school	603 (55.22)	111 (48.47) ^a^	492 (57.01) ^b^	
	Higher education	310 (28.39)	73 (31.88) ^a^	237 (27.46) ^a^	
Body area	Upper extremity	492 (45.05)	131 (57.21) ^a^	361 (41.83) ^b^	<0.001
	Lower extremity	211 (19.32)	45 (19.65) ^a^	166 (19.24) ^a^	
	Torso	350 (32.05)	47 (20.52) ^a^	303 (35.11) ^b^	
	Neck	39 (3.57)	6 (2.62) ^a^	33 (3.82) ^a^	
Time	<1 h	139 (12.73)	11 (4.80) ^a^	128 (14.83) ^b^	<0.001
	1–2 h	196 (17.95)	16 (6.99) ^a^	180 (20.86) ^b^	
	2–3 h	206 (18.86)	39 (17.0) ^a^	167 (19.35) ^a^	
	3–4 h	171 (15.66)	40 (17.47) ^a^	131 (15.18) ^a^	
	4–5 h	143 (13.10)	31 (13.54) ^a^	112 (12.98) ^a^	
	5–6 h	99 (9.07)	40 (17.47) ^a^	59 (6.84) ^b^	
	>6 h	138 (12.64)	52 (22.71) ^a^	86 (9.97) ^b^	
Bleeding		755 (69.14)	175 (76.42)	580 (67.21)	0.007
Stress level	No stress	551 (50.46)	146 (63.76) ^a^	405 (46.93) ^b^	<0.001
	Low	140 (12.82)	28 (12.23) ^a^	112 (12.98) ^a^	
	Medium	300 (27.47)	45 (19.65) ^a^	255 (29.55) ^b^	
	High	101 (9.25)	10 (4.37) ^a^	91 (10.54) ^b^	
Analgesics taken before the procedure		33 (3.02)	9 (3.93)	24 (2.78)	0.367
Cycle phase	Follicular		-	320 (29.30)	-
	Luteal		-	461 (42.22)	
	Menstruation		-	82 (7.51)	

Age, BMI, NRS during tattooing, NRS after tattooing: means (SDs), *p* from the Mann–Whitney U test. Remaining variables: number (percent), *p* from the chi-square test. Different letters (a, b) denote significant gender differences for a given category of the categorical variable at *p* < 0.05.

**Table 2:** Results of regression analyses including gender and excluding cycle phase.

Dependent Variable	Predictors	B	*p*	95% CIs	
NRS during tattooing	Gender	0.05	0.781	−0.33	0.41
	Age	−0.01	0.707	−0.03	0.02
	BMI	−0.01	0.628	−0.04	0.02
	Education	0.25	0.008	0.07	0.43
	Time	0.35	0.001	0.27	0.43
	Bleeding	0.36	0.052	0.00	0.72
	Stress level	0.45	0.001	0.31	0.60
	Analgesics taken prior to the procedure	1.42	0.001	0.60	2.23
	Body area	-	0.094	-	-
NRS after tattooing	Gender	0.62	0.001	0.34	0.90
	Age	−0.01	0.268	−0.03	0.01
	BMI	<0.01	0.932	−0.02	0.02
	Education	0.17	0.032	0.02	0.31
	Time	0.21	0.001	0.15	0.27
	Bleeding	0.47	0.001	0.20	0.72
	Stress level	0.15	0.011	0.04	0.26
	Analgesics taken prior to the procedure	0.42	0.266	−0.29	1.16
	Body area	-	0.742	-	-
Increasing pain	Gender	−0.025	0.726	−0.164	0.126
	Age	−0.004	0.430	−0.015	0.006
	BMI	0.003	0.654	−0.008	0.013
	Education	0.061	0.107	−0.016	0.132
	Time	0.172	0.001	0.142	0.201
	Bleeding	0.152	0.023	0.020	0.281
	Stress level	0.065	0.016	0.013	0.121
	Analgesics taken prior to the procedure	0.097	0.523	−0.218	0.393
	Body area	-	0.886	-	-
Dependent variable	Predictors	OR	*p*	95% CIs	
Pain during tattoing	Gender	−0.14	0.605	−0.65	0.38
	Age	−0.02	0.328	−0.06	0.02
	BMI	0.02	0.386	−0.03	0.06
	Education	−0.07	0.566	−0.27	0.19
	Time	−0.26	0.001	−0.38	−0.15
	Bleeding	−0.62	0.005	−1.05	−0.22
	Stress level	−0.23	0.036	−0.46	−0.02
	Analgesics taken prior to the procedure	−1.12	0.145	−21.39	0.29
	Body area	-	0.730	-	-
Radiating pain	Gender	0.12	0.550	−0.31	0.55
	Age	0.02	0.106	0.00	0.05
	BMI	0.01	0.502	−0.02	0.05
	Education	−0.14	0.169	−0.36	0.05
	Time	−0.24	0.001	−0.33	−0.16
	Bleeding	−0.09	0.619	−0.47	0.27
	Stress level	−0.13	0.113	−0.29	0.03
	Analgesics taken prior to the procedure	−0.39	0.325	−1.16	0.49
	Body area	-	0.012	-	-

B: regression coefficient from linear multiple regression; OR: odds ratio from multiple logistic regression; 95% CIs: bootstrap generated 95% confidence intervals. PLEASE NOTE: regression coefficients (linear regression) and odds ratios (logistic regression) were generated for continuous and binary predictors, respectively. For the categorical predictor ‘Body area’ (four categories), only *p* value was reported, indicating whether the means (for continuous outcomes) or proportions (for binary outcomes) differ significantly among the four groups constituted by the categorical variable.

**Table 3:** Results of regression analyses including cycle phase and excluding gender.

Dependent Variable	Predictors	B	*p*	95% CIs	
NRS during tattooing	Age	−0.01	0.704	−0.04	0.03
	BMI	−0.01	0.704	−0.04	0.03
	Education	0.28	0.004	0.09	0.48
	Time	0.37	0.001	0.27	0.46
	Bleeding	0.22	0.239	−0.15	0.60
	Stress level	0.42	0.001	0.28	0.58
	Analgesics taken prior to the procedure	1.54	0.001	0.58	2.51
	Body area	-	0.323	-	-
	Cycle phase	-	0.691	-	-
NRS after tattooing	Age	−0.02	0.117	−0.04	0.01
	BMI	−0.01	0.683	−0.03	0.02
	Education	0.17	0.037	0.01	0.34
	Time	0.20	0.001	0.13	0.27
	Bleeding	0.45	0.003	0.13	0.74
	Stress level	0.15	0.025	0.03	0.29
	Analgesics taken prior to the procedure	0.64	0.193	−0.33	1.66
	Body area	-	0.642	-	-
	Cycle phase	-	0.975	-	-
Increasing pain	Age	<0.01	0.647	−0.02	0.01
	BMI	<0.01	0.642	−0.01	0.01
	Education	0.05	0.221	−0.03	0.14
	Time	0.19	0.001	0.16	0.22
	Bleeding	0.17	0.021	0.03	0.31
	Stress level	0.05	0.063	0.00	0.11
	Analgesics taken prior to the procedure	0.30	0.026	0.03	0.56
	Body area	-	0.689	-	-
	Cycle phase	-	0.139	-	-
Dependent variable	Predictors	OR	*p*	95% CIs	
Pain during tattoing	Age	0.02	0.415	−0.02	0.07
	BMI	−0.02	0.357	−0.06	0.02
	Education	0.18	0.147	−0.07	0.42
	Time	0.29	0.001	0.17	0.44
	Bleeding	0.49	0.040	−0.01	0.95
	Stress level	0.24	0.033	0.02	0.47
	Analgesics taken prior to the procedure	0.87	0.192	−0.60	21.56
	Body area	-	0.908	-	-
	Cycle phase	-	0.622	-	-
Radiating pain	Age	−0.01	0.015	−0.01	<0.01
	BMI	<0.01	0.979	−0.01	0.01
	Education	0.03	0.124	−0.01	0.07
	Time	0.04	0.001	0.03	0.06
	Bleeding	0.01	0.872	−0.06	0.07
	Stress level	0.02	0.174	−0.01	0.05
	Analgesics taken prior to the procedure	<0.01	0.976	−0.16	0.18
	Body area	-	0.161	-	-
	Cycle phase	-	0.545	-	-

B: regression coefficient from linear multiple regression; OR: odds ratio from multiple logistic regression; 95% CIs: bootstrap generated 95% confidence intervals. PLEASE NOTE: regression coefficients (linear regression) and odds ratios (logistic regression) were generated for continuous and binary predictors, respectively. For the categorical predictors ‘Body area’ (four categories) and ‘Cycle phase’ (three categories), only *p* values were reported, indicating whether the means (for continuous outcomes) or proportions (for binary outcomes) differ significantly among the groups constituted by categorical predictors.
